# Supplementary material for: Chiral Skyrmions Interacting with Chiral Flowers
Source: Nano Lett. 2023 Dec 6;23(24):11793–801. doi: 10.1021/acs.nanolett.3c03792 (PMC10755743; doi:10.1021/acs.nanolett.3c03792)
Supplement: Supplementary file 1 — nl3c03792_si_001.pdf [file nl3c03792_si_001.pdf]

Supporting Information for
"Chiral Skyrmions Interacting with Chiral Flowers"

Xichao Zhang,[1,*] Jing Xia,[2,*] Oleg A. Tretiakov,[3] Motohiko Ezawa,[4] Guoping Zhao,[5] Yan Zhou,[6] Xiaoxi Liu,[2,†] and Masahito Mochizuki[1,‡]

[1]*Department of Applied Physics, Waseda University, Okubo, Shinjuku-ku, Tokyo 169-8555, Japan*

[2]*Department of Electrical and Computer Engineering, Shinshu University, 4-17-1 Wakasato, Nagano 380-8553, Japan*

[3]*School of Physics, The University of New South Wales, Sydney 2052, Australia*

[4]*Department of Applied Physics, The University of Tokyo, 7-3-1 Hongo, Tokyo 113-8656, Japan*

[5]*College of Physics and Electronic Engineering, Sichuan Normal University, Chengdu 610068, China*

[6]*School of Science and Engineering, The Chinese University of Hong Kong, Shenzhen, Guangdong 518172, China*

(Dated: November 20, 2023)

* X.Z. and J.X. contributed equally to this work.

† Email: liu@cs.shinshu-u.ac.jp

‡ Email: masa_mochizuki@waseda.jp

Supplementary Figures

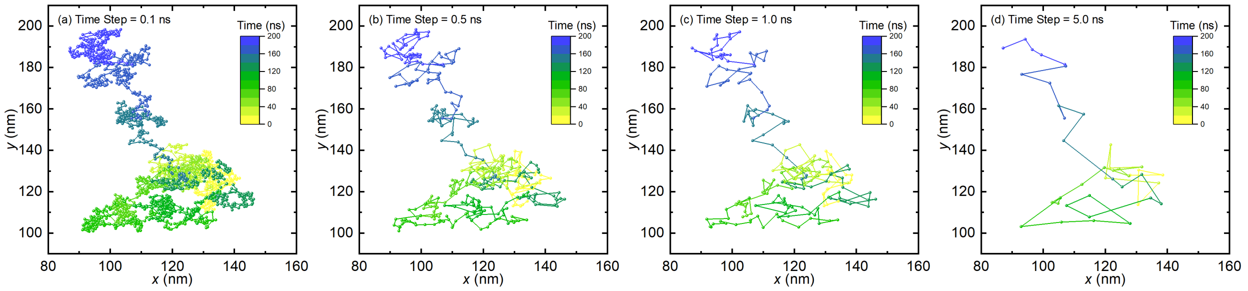

Figure S1. (**a**) Typical random-walk trajectory of a skyrmion with $Q = -1$ in a clean thin film obtained with a time step of 0.1 ns. The skyrmion dynamics is simulated at $T = 150$ K for 200 ns. (**b**) Typical random-walk trajectory of a skyrmion with $Q = -1$ in a clean thin film obtained with a time step of 0.5 ns. The skyrmion dynamics is simulated at $T = 150$ K for 200 ns. (**c**) Typical random-walk trajectory of a skyrmion with $Q = -1$ in a clean thin film obtained with a time step of 1.0 ns. The skyrmion dynamics is simulated at $T = 150$ K for 200 ns. (**d**) Typical random-walk trajectory of a skyrmion with $Q = -1$ in a clean thin film obtained with a time step of 5.0 ns. The skyrmion dynamics is simulated at $T = 150$ K for 200 ns. The above four simulations are done by a workstation with the same random seed. A time step of 0.5 ns is small enough to demonstrate the Brownian motion with a reasonable precision, and meanwhile, can produce an acceptable data size (i.e., 200 data points per 100-ns-long simulation).

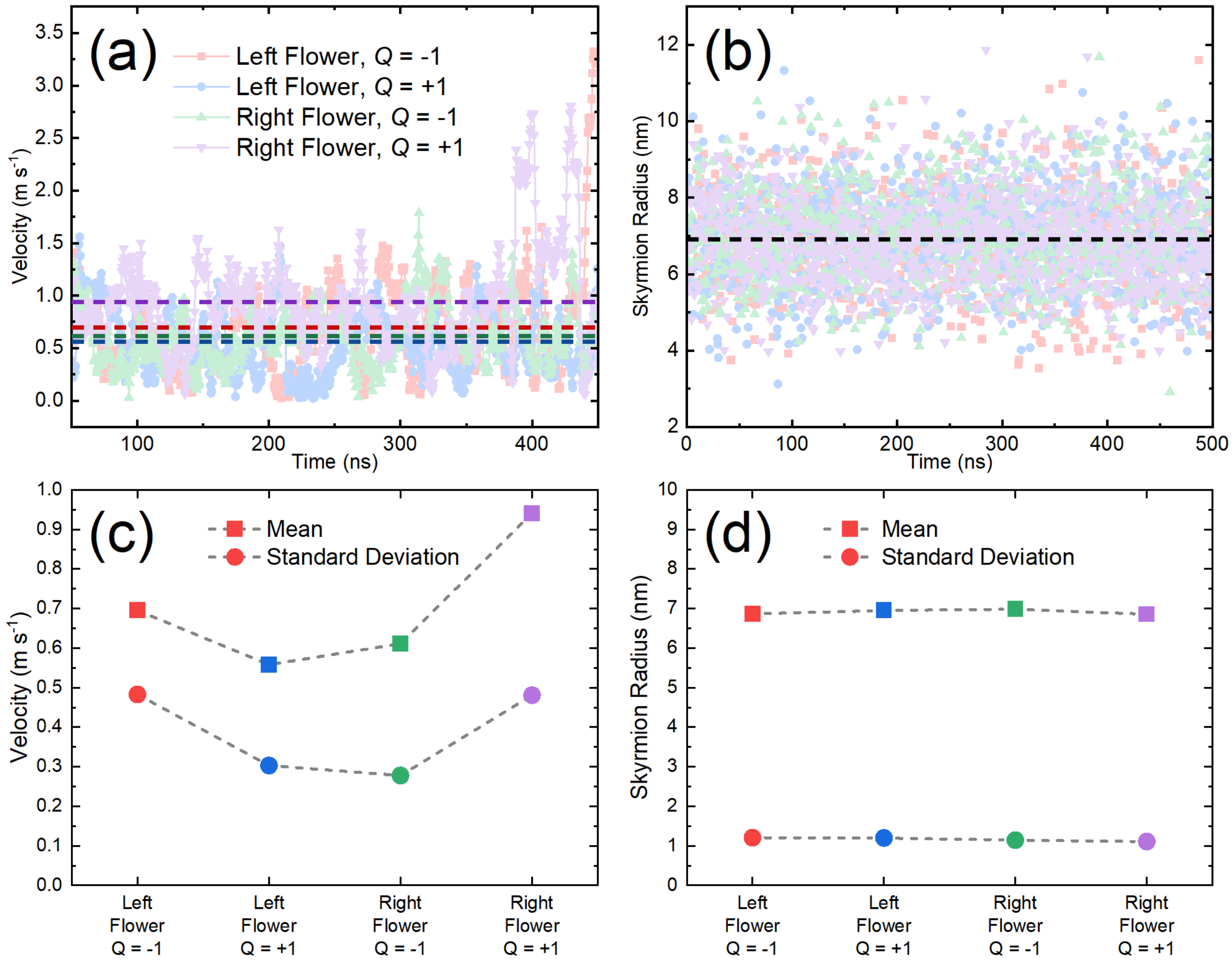

Figure S2. Time-dependent velocity and radius of a skyrmion interacting with a left or right chiral flower. (**a**) Time-dependent velocity of the skyrmion interacting with a left or right chiral flower. (**b**) Time-dependent radius of the skyrmion interacting with a left or right chiral flower. (**c**) Mean value and standard deviation of the skyrmion velocity corresponding to (**a**). (**d**) Mean value and standard deviation of the skyrmion radius corresponding to (**b**). The mean value and standard deviation of the skyrmion velocity could increase when the skyrmion interacts with chiral flower or sample edge more effectively. Therefore, the escaped skyrmion shows slightly increased mean velocity as it can interact with a longer pinning obstacle bar (i.e., the outer edge of the chiral flower) as well as a longer sample edge. However, the variation of the mean skyrmion radius remains tiny. More details are given in the caption of Fig. 3.

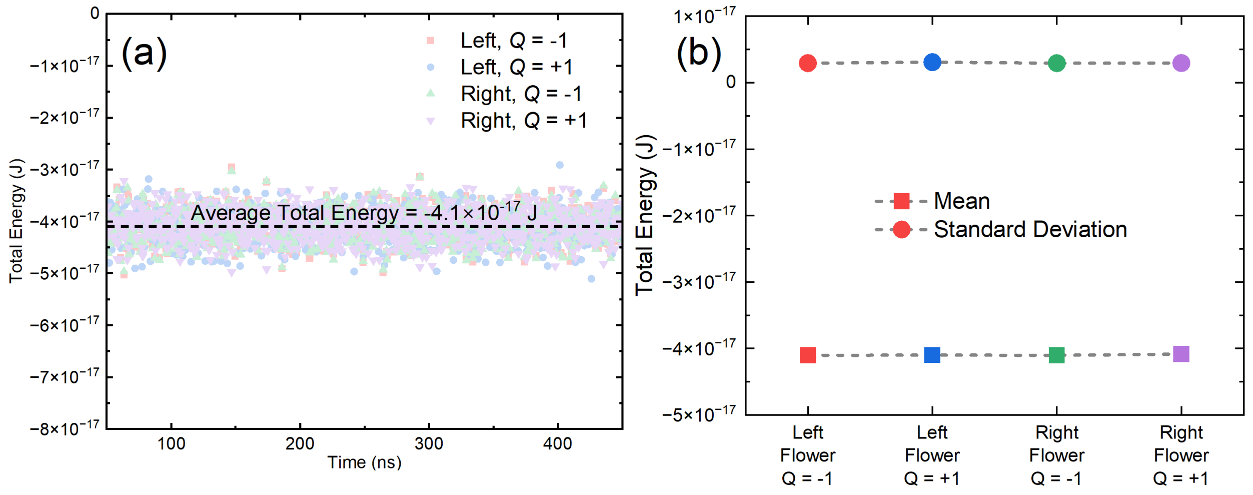

Figure S3. (**a**) Time-dependent total energy of the system, where a skyrmion is interacting with a left or right chiral flower. (**b**) Mean value and standard deviation of the total energy corresponding to (**a**). The total energy of the system is conserved over time despite fluctuations due to the thermal effect. The mean total energy is $\sim -4.1 \times 10^{-17}$ J, and the standard deviation is $\sim 3.0 \times 10^{-18}$ J. More details are given in the caption of Fig. 3.

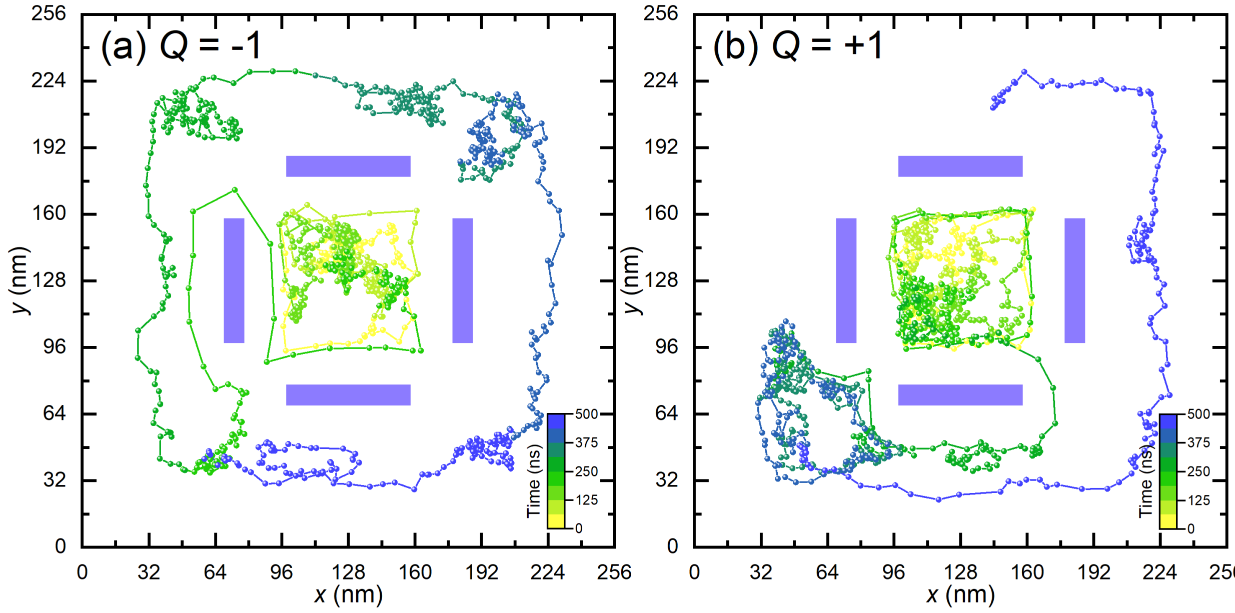

Figure S4. (**a**) Typical trajectory of a skyrmion with $Q = -1$ interacting with an achiral square obstacle with corner gaps. (**b**) Typical trajectory of a skyrmion with $Q = +1$ interacting with an achiral square obstacle with corner gaps. The skyrmion dynamics is simulated at $T = 150$ K for 500 ns with a time step of 0.5 ns. Although the skyrmions with $Q = -1$ and $Q = +1$ could show clockwise and counter-clockwise circular motion in the sample, their interactions with the achiral obstacle lead to trivial outcomes. Namely, both skyrmions with $Q = -1$ and $Q = +1$ can escape easily from the square obstacle, and explore the whole sample.
